# Risk assessment for postoperative outcomes in a mixed hospitalized gynecological population by the Dutch safety management system (Veiligheidsmanagementsysteem, VMS) screening tool ‘frail elderly’

**DOI:** 10.1007/s00404-021-06073-z

**Published:** 2021-04-27

**Authors:** Vera van der Zanden, K. Marieke Paarlberg, Hester J. van der Zaag-Loonen, Wouter J. Meijer, Marian J. E. Mourits, Barbara C. van Munster

**Affiliations:** 1 Department of Obstetrics and Gynecology, Gelre Hospitals, Albert Schweitzerlaan 31, 7334 DZ Apeldoorn, The Netherlands; 2 Department Internal Medicine, University Medical Center Groningen, University of Groningen, Hanzeplein 1, 9713 GZ Groningen, The Netherlands; 3 Department of Obstetrics and Gynecology, University Medical Center Groningen, University of Groningen, Hanzeplein 1, 9713 GZ Groningen, The Netherlands

**Keywords:** Frail elderly, Frailty, Gynecologic surgery, Postoperative complications, VMS, Veiligheidsmanagementsysteem

## Abstract

**Purpose:**

Frailty is associated with a higher risk for negative postoperative outcomes. This study aimed to determine the association between the screening tool of the Dutch safety management system, Veiligheidsmanagementsysteem (VMS) ‘frail elderly’ and postoperative complications in a gynecological population.

**Methods:**

This cohort study included women aged 70 years or older, who were scheduled for any kind of gynecological surgery. VMS screening data (including risk for delirium, falling, malnutrition, and functional impairment) were extracted from the electronic patient records. VMS score could range between 0 and 4 patients with a VMS score of one or more were considered frail. Data on possible confounding factors and complications within 30 days after surgery, classified with the Clavien–Dindo classification, were collected. Regression analysis was performed.

**Results:**

157 women were included with a median age of 74 years (inter quartile range 71–79). Most patients underwent prolapse surgery (52%) or hysterectomy (31%). Forty-one patients (26%) experienced any postoperative complication. Sixty-two patients (39%) were considered frail preoperatively by the VMS screening tool. Frailty measured with the VMS screening tool was not independently associated with postoperative complications in multivariable analysis (Odds ratio 1.18; 95% CI 0.49–2.82). However, a recent fall in the last 6 months (*n* = 208) was associated with postoperative complications (Odds ratio 3.90; 95% CI 1.57–9.66).

**Conclusion:**

An independent association between frailty, determined by the VMS screening tool ‘Frail elderly’, and postoperative complications in gynecological surgery patients could not be confirmed. A recent fall in the last 6 months seems associated with postoperative complications.

## Introduction

Frailty is an important geriatric syndrome and can be defined as a state of increased vulnerability to negative healthcare outcomes after a stressor event due to reduced reserves and function in several systems [1]. Frailty is associated with negative healthcare outcomes, such as postoperative complications, functional decline, loss of independence, lower quality of life, and even death [1–6]. Gynecological problems requiring surgery are common in the older women [7]. Frailty is a common problem with a prevalence ranging between 17% in a non-oncological (measured with Fried criteria [8]) and 25% in an oncological gynecological population (measured with frailty index [9]) [4, 10].

Recent studies in oncological and mixed gynecological populations showed that screening for frailty and adjusting care for frail patients preoperatively can result in better postoperative outcomes [2–4, 11]. A comprehensive geriatric assessment can be used to identify potential modifiable risk factors on several domains. To determine which patients are at risk for frailty and could benefit from a comprehensive geriatric assessment, multiple frailty screening instruments exist. In general gynecology, few frailty screening instruments have been studied yet. The 5-item modified frailty index [12] and the 11-item National Surgical Quality Improvement Program Frailty Index (NSQIP-FI) [2, 13] were associated with postoperative complications in patients undergoing surgery for pelvic organ prolapse [12, 13] or hysterectomy for any indication [2]. Dutch hospitals are obliged by rule and legislation (NTA 8009) [14] to use a screening tool to prevent unnecessary functional decline for all admitted patients aged 70 years and older: the safety management system, Veiligheidsmanagementsysteem (VMS) ‘frail elderly’ [15].

Previous research in various populations showed that the VMS screening tool ‘frail elderly’, is a useful instrument for hospitalized patients to detect frail older patients at risk for adverse outcomes [16–22]. Also, the VMS screening tool was found to be comparable with the Groningen Frailty Index (GFI); paired analysis showed that there was no difference between the two diagnostic tools (*P* = 0.237) [19]. It is unknown whether the VMS screening tool ‘frail elderly’ is a useful instrument in a population of mixed gynecological surgical patients to detect frailty and to predict postoperative complications and mortality. If the tool is found to be associated with negative postoperative outcomes, it could be helpful in pre-operative care, thereby indicating whether there is a need for a comprehensive geriatric assessment and personalized care plan, which could include prehabilitation.

Therefore, the aim of this study was to determine if frailty, as determined by the VMS screening tool ‘frail elderly’, is associated with postoperative complications in gynecological patients. Secondarily, we looked at other postoperative outcomes: postoperative delirium, readmissions, living situation after discharge, and mortality.

## Material and methods

### Study design and setting

This retrospective cohort study was performed using data from the electronic patient records of two general teaching hospitals, Gelre Hospitals, Zutphen and Apeldoorn, the Netherlands.

### Procedures and data assessment

Baseline data were collected from the electronic patient records. Data of the VMS screening tool per item (delirium, falling, malnourishment, and physical status) were also retrieved [15]. This screening instrument is routinely assessed by nurses for all admitted patients aged 70 years and older. In daily practice, the VMS frailty screening tool is not always completed due to the workload in a busy daily clinical practice where nurses might feel less urgency in completing the screening instrument, specifically in cases where a patient looks healthy and fit.

See “Appendix” for the complete VMS screening instrument. A patient is considered at risk for falling if she experienced any fall incident in the last 6 months. A patient is defined to be at risk for delirium if she answers yes to one or more of three questions: memory problems, the need for help with self-care in the last 24 h and the experience of confusion. A patient is considered malnourished if the score on the Short Nutritional Assessment Questionnaire (SNAQ) is ≥ 2 [23]. The six-item Katz Index on independence in activities in daily living (KATZ-ADL6) [24] is used to assess functional status. The cut-off for being dependent is a score of ≥ 2 [15]. Total VMS score was calculated by counting the positive scores on the list of four domains, therefore, the minimum score was zero and the maximum score was four. Being frail was defined as a score of one or more on the VMS screening tool [21].

Data on postoperative complications up until 30 days after surgery, our primary outcome, were classified using the Clavien–Dindo classification [25]. Data on our secondary outcomes: postoperative delirium, readmissions between 48 h after discharge until 3 months after discharge, and living situation after discharge were registered. Information on mortality within 6 months after surgery was retrieved from Dutch Personal Records Database (BRP).

### Participants

Data were included from women who were 70 years or older and had been admitted to the gynecology ward for any kind of gynecological surgical treatment. Inclusion period was between April 2015, which was the start of the routine use of the VMS screening tool in these hospitals, and September 2018. Patients were only included if they had been admitted for 24 h or longer, because only then was the VMS screening tool used. If data of the VMS screening tool were missing, patients in whom at least one positive VMS domain was reported, were included regardless of missing data on the other domains, since frailty was defined as a VMS score of at least one point.

### Statistical analysis

Baseline differences between frail and non-frail patients were compared using a chi-square, an unpaired *T* test or a Mann–Whitney *U* test as appropriate. A *P* value of < 0.05 was considered statistically significant.

The association of the VMS screening tool with the different postoperative outcomes was first evaluated by univariable logistic regression. In the analysis assessing the association between the individual items of the VMS screening tool for postoperative complications, we included those patients for whom the specific item was complete, resulting in different numbers of patients per analysis than the number used for the analysis of the total VMS score.

Due to our small sample size, we only performed multivariable regression analysis to correct for confounders on the primary outcome, postoperative complications. Potentially confounding factors were those variables that were associated with both the outcome and the VMS score or domain (*P* < 0.30). Confounders were included in the model if they altered the regression coefficient of the determinant by more than 10%. All statistical analyses were performed using the statistical package for the social sciences (SPSS), version 25.0.

## Results

**Fig. 1:**
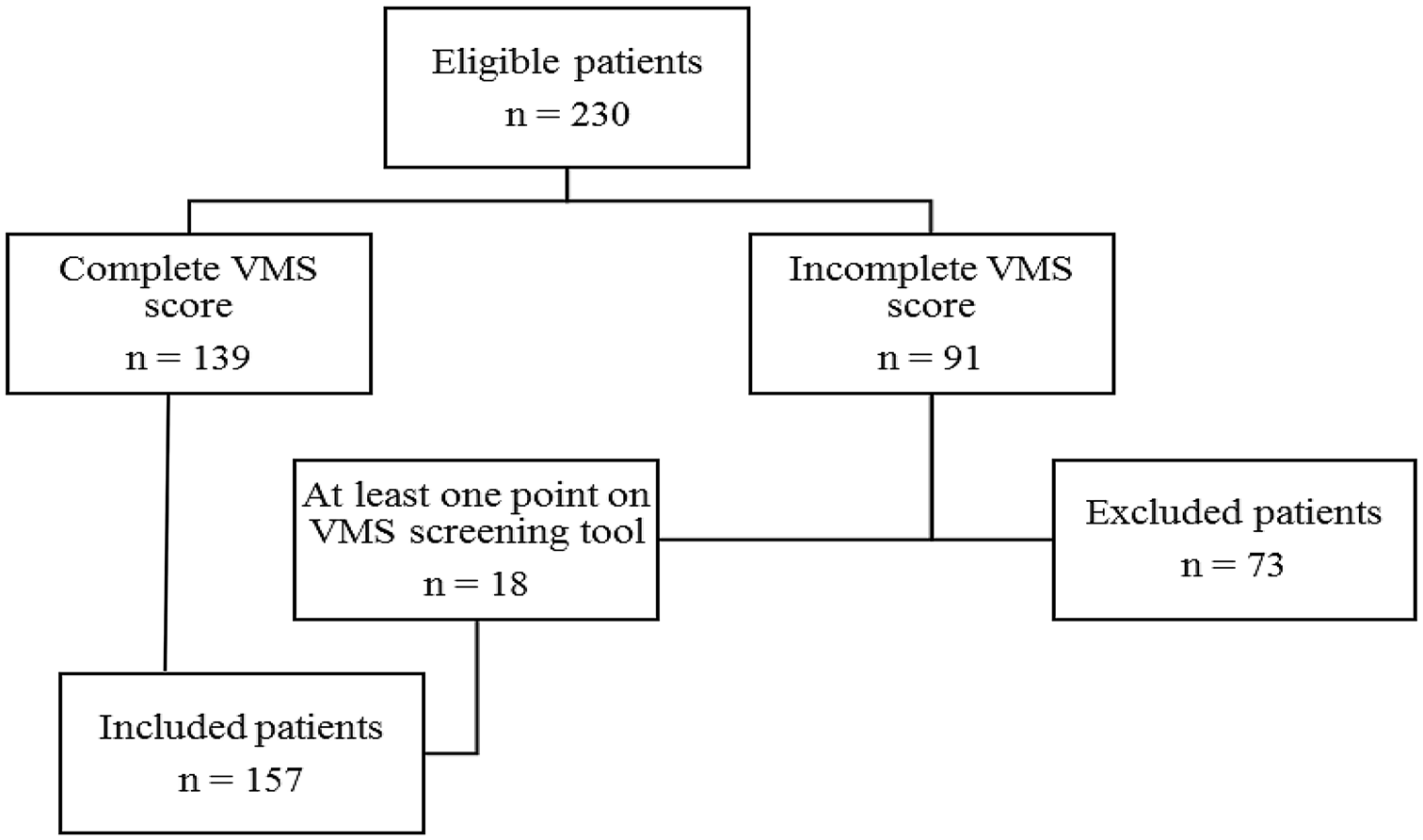
Flowchart of inclusion and exclusion

Data of 157 patients were included in this study. See Fig. 1 for the flowchart of inclusion and exclusion. As compared with the included patients, excluded patients (*n* = 73) had fewer comorbidities (median Charlson Comorbidity Index 0 vs 1, *P* = 0.025) and they less frequently lived in a nursing home (0% vs 3%, *P* = 0.007). Neither included nor excluded patients differed with respect to age (*P* = 0.98), diagnosis (*P* = 0.33), type of operation (*P* = 0.44) or method of surgery (*P* = 0.28), but excluded patients more often received regional anesthesia (29% vs. 17%, *P* = 0.04). The rate of complications was higher in included patients (26% vs. 15%, *P* = 0.06). Table 1 shows the baseline characteristics of the included patients sorted by frailty. The median age of our study population was 74 years, range: 70–94. Frailty was found in 62 patients (39%).

**Table 1:** Characteristics of all included patients

Factor	Study group*				
	VMS score = 0 (*n* = 95)		VMS score ≥ 1 (*n* = 62)		
	Number of patients, unless indicated otherwise	%, Unless indicated otherwise	Number of patients, unless indicated otherwise	%, Unless indicated otherwise	*P* value
Age in years (median; IQR)	74.0	71.0–78.0	76.5	71.8–82.0	**0.005**
**Living situation**					** < 0.001**
Independent at home	94	98.9	40	64.5	
At home with help	1	1.1	17	27.4	
Nursing home facility	0	0.0	5	8.1	
Charlson Comorbidity Index^a^ (median; IQR)	0.0	0.0–1.0	1.0	0.0–2.0	**0.001**
Polypharmacy^b^	39	41.1	44	71.0	** < 0.001**
ASA classification** (***n*** **= 155**)					**0.001**
1	17	17.9	3	4.8	
2	58	61.1	32	51.6	
3	19 (20.0)	20.0	61	41.9	
Smoking	3 (3.2)	3.2	4	6.5	0.62
Use of > 7 units of alcohol per week	9 (9.5)	9.5	10	16.1	0.21
Malignant diagnosis	23 (24.2)	24.2	18	29.0	0.50
**Type of operation**					0.28
Prolapse surgery	45 (47.4)	47.4	36	58.1	
Hysterectomy	34 (35.8)	35.8	14	22.6	
Adnex extirpation	14 (14.7)	14.7	9	14.5	
Vulvectomy	2 (2.1)	2.1	3	4.8	
**Method of surgery**					0.38
Laparotomy	12 (12.6)	12.6	6	9.7	
Laparoscopy	34 (35.8)	35.8	16	25.8	
Vaginal	47 (49.5)	49.5	37	59.7	
Local excision	2 (2.1)	2.1	3	4.8	
General anesthesia	81 (85.3)	85.3	49	79.0	0.31
**VMS score per item**					
At risk for delirium			34	54.8	
Missing			1	1.6	
At risk for falling			22	35.5	
Missing			5	8.1	
SNAQ-score ≥ 2			10	16.1	
Missing			14	22.6	
KATZ-ADL6 ≥ 2			17	27.4	
Missing			6	9.7	

*IQR* inter quartile range, *ASA* American Society of Anesthesiology, *VMS* Veiligheidsmanagementsysteem, *SNAQ* Short Nutritional Assessment Questionnaire, *KATZ−ADL6* six-item Katz Index on independence in activities in daily living

Boldface data are statistically significant

*Number (%) of patients, unless indicated otherwise

**The American Society of Anesthesia Classification (measured before surgery) ranges from 1 to 6, with higher scores indicating worse physiological status and a higher operative risk [34]

^a^The Charlson Comorbidity Index ranges from 1 to 31, with higher scores indicating more comorbidities [32]

^b^Polypharmacy was defined as the use of ≥ 5 different medicines (ATC level 3), dermatological medicines (creams, ointments etc.) excluded [33]

Table 2 shows the descriptive statistics of all outcome variables sorted by frailty. Postoperative complications were found in 41 patients (26%). Most patients had a complication directly related to surgery (*n* = 33; 21%). Six patients (4%) had a cardiopulmonary complication, one patient (1%) had both a surgical and a cardiopulmonary complication, and one patient (1%) suffered from both a surgical complication and a postoperative delirium. Surgical complications consisted mostly of urinary retention (*n* = 12; 8%) or a urinary tract infection (*n* = 7; 5%). Furthermore, surgical complications were persistent pain (*n* = 6, 4%), blood loss (*n* = 3, 2%), wound infections (*n* = 2, 1%), or other complications (*n* = 5, 3.2%). When grading the complications using the Clavien–Dindo classification, 23 patients (15%) had a Clavien–Dindo grade I complication, 12 (8%) a grade II complication, 5 (3%) a grade III complication, and 1 (1%) a grade IV complication. There was no difference in the incidence of overall complications (24.7% vs. 16.1%; *P* = 0.18) or severe complications between patients with a benign or malignant diagnosis (2.9% vs. 3.6%; *P* = 0.79). None of the patients died after surgery. One patient died within 90 days of surgery, not related to the operation, no other patients died within 6 months after surgery.

**Table 2:** Descriptive statistics of primary and secondary outcomes

Factor	Study group* % of patients, unless indicated otherwise		
	VMS score = 0 (*n* = 95)	VMS score ≥ 1 (*n *= 62)	*P* value
Any complication within 30 days after surgery	20.0	35.5	**0.03**
Severe complications^a^	2.1	6.5	0.17
Mortality within 90 days after discharge	0.0	1.6	0.21
Duration of admission in days (median; IQR)	2.0 (1.0–2.0)	2.0 (1.0–3.0)	**0.02**
Readmissions within 30 days after discharge	2.1	4.8	0.34

Boldface data are statistically significant

IQR inter quartile range

*Number (%) of patients, unless indicated otherwise

^a^Clavien−Dindo > 2

Table 3 shows the regression analysis of the VMS score and the association with postoperative outcomes. With univariable logistic regression, we found that being frail was associated with postoperative complications within 30 days after surgery (Odds ratio 2.20; 95% CI 1.07–4.54). In multivariable analysis the association decreased (Odds ratio 1.18, 95% CI 0.49–2.82). Table 4 shows the individual association of the individual domains of the VMS screening tool with postoperative complications. Being at risk for falling was independently associated with postoperative complications within 30 days after surgery.

**Table 3:** Results from univariable and multivariable analyses, association with any postoperative complication within 30 days after surgery (*n* = 157)

Outcomes	Odds ratio	95% CI	*P* value	Odds ratio	95% CI	*P* value
	Univariable analysis			Multivariable analysis		
VMS score ≥ 1	2.20	1.07–4.54	**0.03**	1.18	0.49–2.82	0.72
Age	1.07	1.00–1.13	**0.05**	1.03	0.96–1.11	0.39
Polypharmacy	3.82	1.71–8.50	**0.001**	2.94	1.25–6.92	**0.013**
Living situation	2.86	1.37–3.94	**0.005**	1.67	0.71–3.94	0.24

Bold face data are statistically significant

*VMS* Veiligheidsmanagementsysteem

**Table 4:** Results from univariable and multivariable analyses, associations of the individual items of the VMS screening tool with postoperative complications within 30 days after surgery

Outcomes	Odds ratio	95% CI	*P* value	Odds ratio	95% CI	*P* value
	Univariable analysis			Multivariable analysis		
At risk for delirium (*n* = 215)	2.05	0.93–4.5	0.07			
At risk for falling (*n* = 208)	3.90	1.57–9.66	**0.003**	3.90^a^	1.57–9.66	**0.003**
SNAQ-score ≥ 2 (*n* = 159)	0.73	0.15–3.59	0.70			
KATZ-ADL6 ≥ 2 (*n* = 191)	3.18	1.15–8.80	**0.03**	1.31^b^	0.35–4.90	0.69

*VMS* Veiligheidsmanagementsysteem, *SNAQ* short nutritional assessment questionnaire, *KATZ−ADL6* six-item Katz Index on independence in activities in daily living

Boldface data are statistically significant

^a^We considered age, polypharmacy, living situation and method of surgery as potential confounders (*P* < 0.30). In the multivariable model, none of these appeared to be confounders to adjust for

^b^Odds ratio adjusted for polypharmacy and living situation

## Discussion

Our study concludes that being frail, according to a VMS score of one ore more, was not significantly associated with postoperative complications within 30 days after surgery, but a recent fall was significantly associated with postoperative complications within 30 days after surgery. Falling is an important geriatric syndrome, and is more prevalent in patients with sarcopenia [26]. Since falling, among others, could be an utterance of sarcopenia and sarcopenia is associated with postoperative complications [26], it is understandable that falling is associated with postoperative complications as well. A recent meta-analysis in cancer patients found that in less than half of the included studies, an association between falling and postoperative complications and mortality was found [27]. Studies performed in a non-solely oncological population showed that a history of one or more falls in the 6 months prior to an operation forecasts negative healthcare outcomes [28, 29]. Our study indicates that, besides attention for fall risk reduction [30], caution for postoperative complications is needed if a patient reports any fall in the previous 6 months.

In contrast to our study, other surgical studies using the VMS score in abdominal [21] and hip fracture surgery [19] showed that the VMS frailty screening tool was independently predictive for postoperative outcomes, such as overall complication rate [21] and survival [19, 21]. Several arguments for our different findings can be brought forward.

First, differences in study population, type of complications explored or VMS cut-off point used may explain our findings. Most patient in our population underwent low-risk surgery. Furthermore, it could be possible that non-surgical complications, such as cardiopulmonary complications or thromboembolic complications, in specific are more related to comorbidity and, therefore, more associated with frailty. Souwer et al. showed a relation between the VMS and complication occurrence, but not with surgical complications [21]. They found a lower percentage of surgical complications (46% of all complications) than in our population (81% of all complications).

Second, it is possible that a higher cut-off point is more associated with the outcomes than our cut-off point of one. In the previous studies, higher scores were more strongly associated with complications [19, 21]. Using a different cut-off point or creating groups with increasing frailty (e.g., sum scores 0, 1–2, 3–4) like Souwer et al. did, was not possible in our study since in our population few patients scored higher than one. Besides that, summing the different domains to get one score may be less accurate than looking at the different domains separately. As we found in our study, falling was associated with postoperative complications, but the other domains were not.

Lastly, since there are very few studies on this subject, the association of the VMS screening tool with postoperative outcomes is not established yet. It is possible that the association between the VMS screening tool and postoperative complications is not as strong as the current evidence suggests, because aspects like publication bias may have distorted the true association.

Different versions of the frailty index were associated with postoperative complications in gynecological patients [2, 12, 13]. The population in the study of George et al. is the most comparable to our population, because it includes both non-oncological and oncological patients as well. They calculated an 11-item modified frailty index and found it to be associated with complications and mortality [2]. But also in the low-risk population of patients undergoing prolapse surgery, frailty measured with the frailty index was associated with worse postoperative outcomes [12, 13]. Therefore, we can conclude that frailty is a problem in a general gynecological surgery population, only the VMS screening tool seems to be not suitable to detect it properly in this group.

## Strengths and limitations

Our study has several strengths and limitations. The strength of this study is that we used wide inclusion criteria, resulting in a representative cohort of Dutch older gynecological surgery patients in two general teaching hospitals. To our knowledge, no previous studies investigated the association of VMS frailty scores with postoperative outcomes in gynecological patients.

There are some limitations to our study as well. Because of the retrospective nature of the study, outcome parameters were limited to the ones that could be collected from the electronic patient records. While most older patients are more interested in these functional outcomes, such as maintaining independence, these patient-related functional outcomes could not be collected [31]. Furthermore, because data were missing not at random (MNAR), we analyzed a small and relatively frail subgroup of the total population of gynecological patients. Our results reflect daily clinical practice in general hospitals, since only complete VMS scores of a selected group will be available in clinical practice as well. As mentioned before, our sample size was 157. An association between the VMS frailty screening tool and postoperative complications might have been demonstrated in a larger population. However, if we need more patients to demonstrate any association, the clinical relevance for daily practice is limited.

## Conclusion

We were not able to demonstrate an independent association between the VMS screening tool ‘frail elderly’ and postoperative complications in general gynecological surgery patients. Any patient fall in the last 6 months prior to surgery, however, is associated with postoperative complications. Our study implies that caution is needed if a patient reports a fall in the previous 6 months and a consultation with a geriatrician should be considered.

With an increasingly ageing population worldwide, more knowledge is needed on the impact of surgery, on how to identify the patients most at risk, and how to care for older gynecological surgery patients. A reliable screening instrument for frailty in the selection of patients for pre-operative optimization and geriatric co-management before, during and after hospitalization is needed. The VMS screening tool is not the instrument of choice.

## Data Availability

Not applicable.
